# Primary choriocarcinoma of the posterior mediastinum in a male: A case report and review of the literature

**DOI:** 10.3892/ol.2014.2222

**Published:** 2014-06-04

**Authors:** FANGBIAO ZHANG, WEIDONG ZHANG, HONGCAN SHI, GANG YE, WEIPING SHI, YUSHENG SHU, GUANGYU LI

**Affiliations:** Department of Cardiothoracic Surgery, Clinical College, Yangzhou University, Yangzhou, Jiangsu 225001, P.R. China

**Keywords:** posterior mediastinum, chemotherapy, surgery, choriocarcinoma

## Abstract

Primary choriocarcinoma of the posterior mediastinum is considered to be extremely rare, and the majority of these tumors occur in the gonads and uterus. The current study presents the case of a 40-year-old male who presented to the Department of Thoracic Surgery (Medical College of Yangzhou University, Yangzhou, China) with left chest pain for two months. Computed tomography of the chest showed a 4.9×5.2-cm mass in the posterior mediastinum and enlargement of the mediastinal lymph nodes. Resection of the tumor and upper lobe of the left lung was performed and the patient received combination chemotherapy with six courses of etoposide plus cisplatin. The patient recovered and was discharged. One year post surgery and chemotherapy, the patient was followed up and evidence of a recurrent tumor in the cerebrum was observed.

## Introduction

Primary choriocarcinoma is an unusual neoplasm of the posterior mediastinum, which frequently occurs in the gonads and uterus, and less frequently in the extragonadal sites (1). There are several theories regarding the development of primary mediastinal choriocarcinoma, including abnormal germ migration (2) and tumor cell distortion (3). However, the precise genetic mechanism remains unknown. The majority of cases are misdiagnosed by imaging examination and clinical features prior to surgery (1). Chemotherapy, radiotherapy and surgery are the main therapeutic strategies, however, the effect of any method of treatment is poor. The current study presents a case of primary choriocarcinoma of the posterior mediastinum in a 40-year-old male and reviews the characteristics of previously reported cases. The aim of this report is to explore the diagnosis and treatment of primary choriocarcinoma of the posterior mediastinum.

## Case report

**Figure 1:**
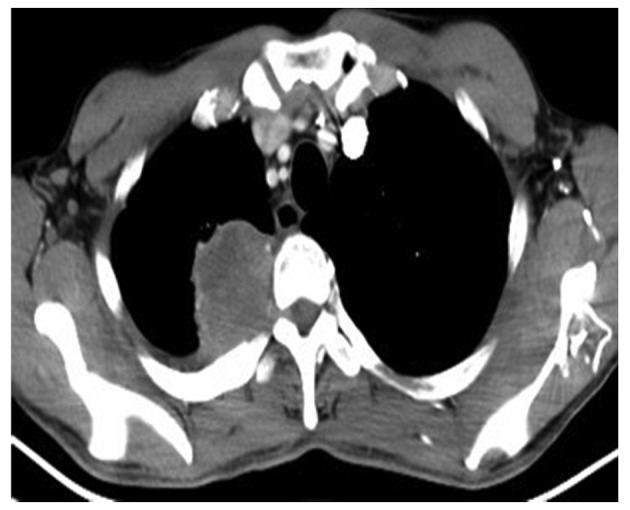
Computed tomography of the chest showed a large mass in the posterior mediastinum.

**Figure 2:**
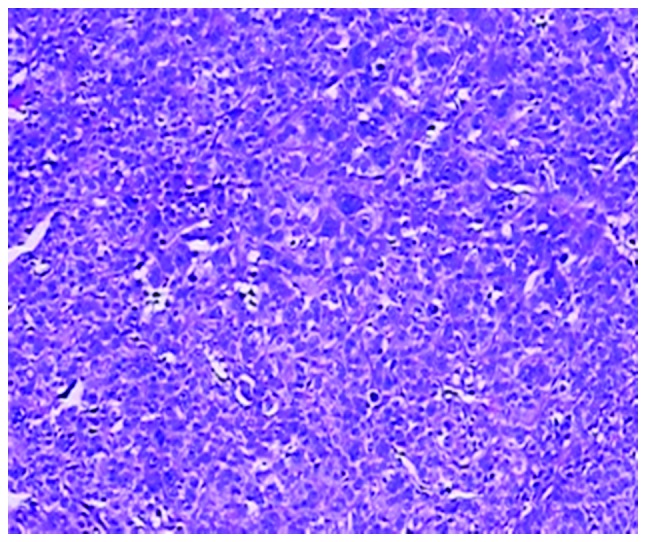
Pathological examination showed magnocellular, dark-stained and pleomorphic nuclei (hematoxylin and eosin stain; magnification, ×100).

**Figure 3:**
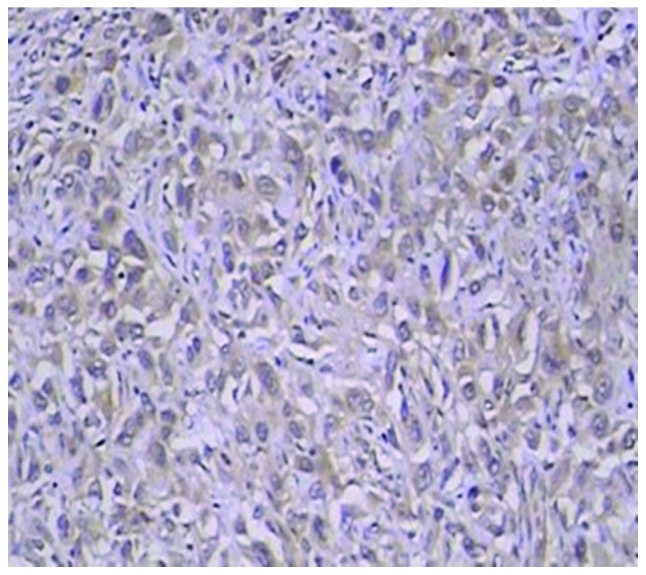
Immunohistochemical reaction for β-human chorionic gonadotropin was positive (immunohistochemical staining with human chorionic gonadotropin (HCG), s-100, cytokeratin 5, CD56, syn, cytokeratin 7 and NapsinA; magnification, ×100).

**Figure 4:**
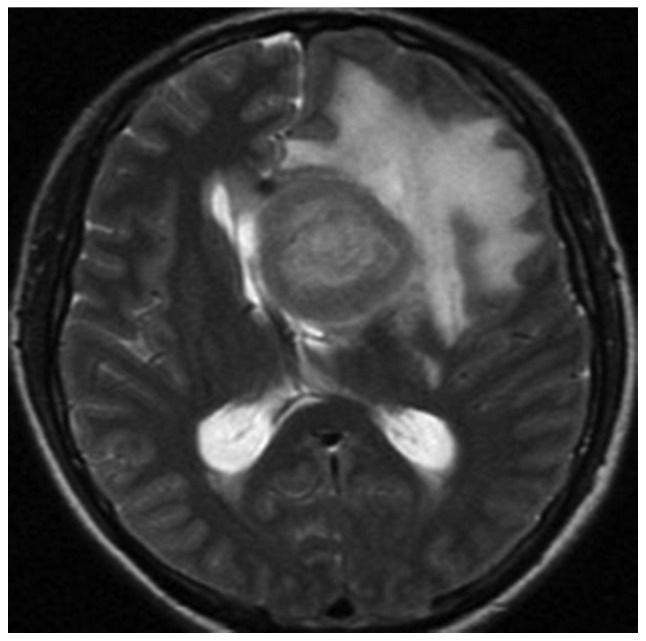
Computed tomography of the cerebrum after one year of follow-up showed a large mass in the cerebrum.

A 40-year-old male presented to the Department of Thoracic Surgery (Medical College of Yangzhou University, Yangzhou, China) with left chest pain, which had endured for two months. Computed tomography (CT) showed a 4.9×5.2-cm mass in the posterior mediastinum and enlargement of the mediastinal lymph nodes, while no other lesions were identified in the thoracic cavity (Fig. 1). A CT-guided percutaneous aspiration of the mediastinal mass led to a diagnosis of adenocarcinoma. Due to the diagnosis of a malignant tumor, the patient underwent surgical removal of the entire tumor. After providing written informed consent, the patient underwent surgical intervention on March 5, 2012. During surgery, the left upper lung lobe was found to be invaded and therefore, the tumor and upper lobe of the left lung was dissected. Simultaneously, lymph node dissection was performed, which involved radical dissection of the mediastinum. Following surgery, pathological examination revealed a malignant tumor with a diffuse distribution of abnormal cells, with magnocellular, dark-stained and pleomorphic nuclei (Fig. 2). The immunohistochemical reaction for β-human chorionic gonadotropin (hCG) was positive, and a fine, granular and brown deposit was observed within the cytoplasm of multinucleate giant cells in all areas of the sampled tumor (Fig. 3). Histological examination and immunohistochemical staining of the surgical specimens confirmed choriocarcinoma with invasion of the upper lobe of the left lung. The patient received combination chemotherapy with six courses of etoposide plus cisplatin. The preoperative serum concentration of β-hCG was 1.0 mIU/ml (normal range, <100 mIU/ml). The abdominal CT was unremarkable and the testes appeared to be normal on the ultrasound. Following the six cycles of chemotherapy, the patient recovered and was discharged. However, one year post surgery and chemotherapy, the patient was followed up and evidence of a recurrent tumor in the cerebrum was observed (Fig. 4).

## Discussion

Choriocarcinoma is a highly malignant trophoblastic tumor, which commonly arises in pregnant females, with a few cases arising in females and males also being reported. Early metastasis may occur via blood circulation throughout the body, which results in bleeding and necrosis of tissues and organs. Primary choriocarcinoma of the posterior mediastinum in males is particularly rare and treatment is considered to be ineffective. As choriocarcinoma grow rapidly, early recurrence and metastasis are frequently observed. Common symptoms include coughing, fever, chest pain and breathing difficulties. The histological features of choriocarcinoma are characterized by a biphasic cellular population with extensive areas of hemorrhage and necrosis (4). There are two types of cells in this tumor; cytotrophoblastic and syncytiotrophoblastic. The cytotrophoblastic type is characterized by round-to-polygonal cells with clear, polyhedral cytoplasm, round nuclei, sparse chromatin and prominent nucleoli, while the syncytiotrophoblastic type is composed of polynuclear giant cells with abundant eosinophilic cytoplasm (4).

The immunohistochemical reaction for β-hCG is the predominant method of diagnosis for choriocarcinoma. In addition, developments in technology and advanced equipment, such as 18F-fluorodeoxyglucose positron emission tomography (FDG-PET), can be used to assess the diagnosis and prognosis of this type of tumor. Shukuya *et al* (5) reported a case of an elderly male with primary mediastinal choriocarcinoma, who underwent surgery and four cycles of chemotherapy. Following treatment, specific sections of the residual tumors on the CT images showed markedly reduced uptake, while the other sections showed similar uptake in FDG-PET. Therefore, FDG-PET may facilitate the evaluation of the viability of tumors following treatment in cases of mediastinal choriocarcinoma.

Numerous studies on extragenital choriocarcinomas occurring in other organs, such as the lung (6), stomach (7) and colon (8), have previously been reported. To the best of our knowledge, the first description of a posterior mediastinal choriocarcinoma was by Yamashita *et al* (9) in 2002 of a 29-year-old male with posterior mediastinal choriocarcinoma. The patient received two cycles of combination chemotherapy. However, the patient succumbed to the disease approximately three months following the diagnosis. A search was conducted using the electronic database, PubMed, for all the relevant literature between 2002 and 2013, involving primary choriocarcinoma of the mediastinum. Since 2002, no similar neoplasms occurring in the posterior mediastinum in males have been reported.

The prognosis for patients with mediastinal choriocarcinoma appears to be poor (9). Lynch and Blewitt (10) reported a case of a 26-year-old male who succumbed to the disease two weeks following the diagnosis of choriocarcinoma; the total duration of the illness had been six weeks. Ramia *et al* (11) presented the first case of a patient that presented with appendicular metastasis from a choriocarcinoma of the mediastinum who underwent an appendectomy and radical surgery of the mediastinal tumor. However, during chemotherapy the patient showed progressive clinical deterioration and succumbed to the disease five months following the appendectomy. Certain studies have reported long-term survival. Kathuria *et al* (12) reported a case of mediastinal choriocarcinoma where the patient underwent surgery in association with chemotherapy and at the two-year follow-up, no lymphadenopathy was observed and the other physical observations were unremarkable. In addition, Zheng (13) reported a case of a young male with primary anterior mediastinal choriocarcinoma that was accompanied by primary cystoid teratoma. The patient underwent surgery in combination with chemotherapy and survived for at least one year. According to the study by Moran *et al* (4), no patient survived for more than one year; however, one patient survived for one year, although, a brain tumor, which was potentially metastatic, was identified. The level of β-hCG is considered to be a major factor affecting the prognosis of primary choriocarcinoma of the posterior mediastinum. The treatment of patients with a combination of surgery combined with chemotherapy is likely to improve the long-term outcome (14).

In conclusion, this study presents an extremely rare case of a patient with primary mediastinal choriocarcinoma, who underwent successful surgery and chemotherapy. The patient recovered and was discharged with no issues. Thus, the early diagnosis and timely treatment of primary mediastinal choriocarcinoma is key to improving the long-term outcome.

## References

[1] Shi H, Cao D, Wei L, Sun L, Guo A (2010). Primary choriocarcinoma of the liver: a clinicopathological study of five cases in males. Virchows Arch.

[2] Sullivan LG (1989). Primary choriocarcinoma of the lung in a man. Arch Pathol Lab Med.

[3] Pushchak MJ, Farhi DC (1987). Primary choriocarcinoma of the lung. Arch Pathol Lab Med.

[4] Moran CA, Suster S, Koss MN (1997). Primary germ cell tumors of the mediastinum: III. Yolk sac tumor, embryonal carcinoma, choriocarcinoma, and combined nonteratomatous germ cell tumors of the mediastinum - a clinicopathologic and immunohistochemical study of 64 cases. Cancer.

[5] Shukuya T, Hirano S, Takeda Y, Ito H, Hurihata K, Sugiyama H, Kobayashi N, Kudo K (2007). A case of primary mediastinal choriocarcinoma in which FDG-PET was performed for the evaluation of the treatment. Nihon Kokyuki Gakkai Zasshi.

[6] Ibi T, Hirai K, Bessho R, Kawamoto M, Koizumi K, Shimizu K (2012). Choriocarcinoma of the lung: report of a case. Gen Thorac Cardiovasc Surg.

[7] Kobayashi A, Hasebe T, Endo Y, Sasaki S, Konishi M, Sugito M, Kinoshita T, Saito N, Ochiai A (2005). Primary gastric choriocarcinoma: two case reports and a pooled analysis of 53 cases. Gastric Cancer.

[8] Le DT, Austin RC, Payne SN, Dworkin MJ, Chappell ME (2003). Choriocarcinoma of the colon: report of a case and review of the literature. Dis Colon Rectum.

[9] Yamashita S, Ohyama C, Nakagawa H, Takeuchi A, Watanabe M, Hoshi S (2002). Primary choriocarcinoma in the posterior mediastinum. J Urol.

[10] Lynch MJ, Blewitt GL (1953). Choriocarcinoma arising in the male mediastinum. Thorax.

[11] Ramia JM, Alcalde J, Dhimes P, Cubedo R (1998). Metastasis from choriocarcinoma of the mediastinum producing acute appendicitis. Dig Dis Sci.

[12] Kathuria S, Jablokow VR (1987). Primary choriocarcinoma of mediastinum with immunohistochemical study and review of the literature. J Surg Oncol.

[13] Zheng XM (2001). Primary cystoid teratoma companied with primary choriocarcinoma in anterior mediastinum: a case report. J Diagn Pathol (Chin).

[14] Shen HH, Zhang GS, Xu F (2004). Primary choriocarcinoma in the anterior mediastinum in a man: a case report and review of the literatures. Chin Med J (Engl).

